# Gender inequality in self-reported health among the elderly in contemporary welfare countries: A cross-country analysis of time use activities, socioeconomic positions and family characteristics

**DOI:** 10.1371/journal.pone.0184676

**Published:** 2017-09-26

**Authors:** Nicholas Kofi Adjei, Tilman Brand, Hajo Zeeb

**Affiliations:** 1 Leibniz Institute for Prevention Research and Epidemiology - BIPS, Bremen, Germany; 2 Health Sciences Bremen, University of Bremen, Bremen, Germany; University of Michigan, UNITED STATES

## Abstract

**Background:**

Paradoxically, despite their longer life expectancy, women report poorer health than men. Time devoted to differing social roles could be an explanation for the observed gender differences in health among the elderly. The objective of this study was to explain gender differences in self-reported health among the elderly by taking time use activities, socio-economic positions, family characteristics and cross-national differences into account.

**Methods:**

Data from the Multinational Time Use Study (MTUS) on 13,223 men and 18,192 women from Germany, Italy, Spain, UK and the US were analyzed. Multiple binary logistic regression models were used to examine the association between social factors and health for men and women separately. We further identified the relative contribution of different factors to total gender inequality in health using the Blinder-Oaxaca decomposition method.

**Results:**

Whereas time allocated to paid work, housework and active leisure activities were positively associated with health, time devoted to passive leisure and personal activities were negatively associated with health among both men and women, but the magnitude of the association varied by gender and country. We found significant gender differences in health in Germany, Italy and Spain, but not in the other countries. The decomposition showed that differences in the time allocated to active leisure and level of educational attainment accounted for the largest health gap.

**Conclusions:**

Our study represents a first step in understanding cross-national differences in the association between health status and time devoted to role-related activities among elderly men and women. The results, therefore, demonstrate the need of using an integrated framework of social factors in analyzing and explaining the gender and cross-national differences in the health of the elderly population.

## Introduction

Over the past decades, population ageing has been one of the major global demographic processes [1–3]. The percentage of those aged 60 years and above increased from 8% in 1950 to 12% in 2013 and it is projected to increase to 21% by 2050 [4]. Empirical research shows that women have a longer life expectancy than men [5–7]. In 2013, UN data indicated that 85 men per 100 women were 60 years or over and 61 men per 100 women were 80 years or over [4]. Although women live longer than men, they report poorer health [8], as well as more physical limitation [9] and chronic conditions [10]. This health-survival paradox can partly be explained by gender differences in biological factors such as genetics and hormonal exposure [11–13]. Several earlier studies have also shown that gender differences in socio-economic position (SEP) contribute to health disparities among the younger population [14–16] and the elderly [17–19]. A possible explanation of this outcome is that SEP is often lower among women and thus they are exposed to more health adversities [20]. However, there is still no consensus about the best indicators of SEP to be used among the elderly [19,21–23]. Thus, there is a need to further explore the suitability of reliable social factors among older men and women.

Apart from biological factors and SEP, social roles and activities may explain gender differences in health [24]. Since gender is perceived to be a distinct feature with respect to social roles, some studies have examined the differences in time spent on role-related activities among men and women [25,26]. Although men have increased the amount of time allocated to some role-related activities such as housework, their contribution to these activities remains lower than that of women [25]. Coltrane [26] showed that women spend two or three times more time doing routine, repetitive housework than men. Even after retirement, gender roles are still shaped in a traditional way in some welfare countries, especially in the Southern European countries, where women continue to assume the role of a housewife [27]. This unequal distribution of household activities limits women’s participation in active leisure and other social activities [28], which may have a negative effect on their health.

However, the extent of gender and cross-national differences in the distribution of time regarding role-related activities varies by social norms and national policies [27,29]. These mediating factors have also been identified as potential contributing factors to health inequality. For example, Eikemo and colleagues [30] found that 10 percent of differences in self-reported health could be linked with welfare states characteristics. Thus, policies and social norms may affect the allocation of time by influencing the patterns of daily activities.

Several studies have explored the relationship between social roles and health [31–33], but only some have focused on this topic among the elderly [34,35]. Moreover, the conceptual framework used by these studies on the elderly was related to “role occupancy”, such as parental status (i.e., the presence of children in the household) and marital status (i.e., being married, divorced, separated or widowed), and their associations with health. These measures of social roles are crude and indirect and might give little information on how much time and effort are spent on role-related activities such as housework, childcare, and other household activities [24].

In this study, we operationalized social roles as time allocation to the various role-related activities among older men and women based on Bird and Fremont [24]. Time use data was used to examine the extent to which the “role occupant” fulfils the role. The amount of time spent on role-related activities such as household work, childcare, maintenance, voluntary work and other activities were estimated using diary-based time allocation data. Time diary has been shown to be more reliable, accurate and providing a better picture of how social roles influence health as compared to survey estimates [24].

So far, only four studies have examined the relationship between time allocation and health [24,36–38]. Time allocated to differing social roles has yet to be examined as an explanation for the observed gender differences in health among the elderly. The objective of this study is to explore social and economic determinants of health among elderly men and women simultaneously, using a combined framework of time use activities, socio-economic positions and family characteristics. The study aims to explain the gender and cross-national differences in health based on data from five welfare countries (Germany, Italy, Spain, United Kingdom and the United States).

The following research questions will be addressed:

1a) How do time use activities, SEP and family characteristics impact the health among the elderly?
b) To what extent do these effects vary by gender and across countries.
c) To what extent do these social factors explain gender differences in health among the elderly.

## Methods

### Data

We used data from the Multinational Time Use Study (MTUS, version W53). The MTUS data is a large cross-national, harmonized and comparative time-use database from 25 countries across six waves. This data collection has been organized by the Centre for Time Use Research, located in the Department of Sociology at the University of Oxford. The data set contains information on the socio-economic and demographic background of the respective diarist and the total time spent on 41 activities over a 24-hour period [39]. The full-day period diaries were self-administered, followed by a personal visit of study staff in most European countries. The authors were granted approval from the Multinational Time Use Study Review Board to obtain and use the collected data for analysis. All data were anonymized prior to the authors receiving the data.

For the purpose of this study, we limited our sample set to respondents who were 65 years and above at the time of study. The minimum age was chosen based on the retirement age in most EU countries [40]. We also included information from retired persons who had paid work. The countries included in this analysis are the United Kingdom (survey year, 2000); the United States (survey year, 2003); Spain (survey year, 2002); Italy (survey year, 2002); and Germany (survey year, 2001). Data from these countries were of special interest because they include the outcome variable “self-reported health” and numerous independent variables relevant for this study. The use of data from multiple countries furthermore allows for direct comparison of findings.

### Health outcome

The study used self-reported health as a measure of health status (“How is your health in general; would you say that it is ….?” response options: zero (poor) to three (very good)). We created a dichotomous outcome as in [41], where good health took a value of “0” if the respondent reported “very good” or “good” health and a value of “1” if they reported “poor” or “fair” health.

### Time use

All time use variables were measured in hours per day. We limited our study to respondents who reported all 1440 minutes (24 hours) of activities during the day in the diary, and hence adopted the broad categories suggested earlier by Gauthier and Smeeding [1]. S1 Table (appendix) lists the detailed activities included in the following 5 broad categories.

- Paid work (e.g. paid work, travel to and from work)
- Housework (e.g. cooking, washing, gardening, shopping)
- Active leisure Activities (e.g. walking, volunteer, sports, travel for pleasure)
- Personal activities (e.g. sleep, eating, bathing, dressing, medical care)
- Passive leisure activities (e.g. watching television, relaxing)

### Socio-economic position and family characteristics

Socio-economic positions were measured by three indicators: Education, wealth and employment status. Education was categorized into three groups: less than secondary education, completed secondary education and above secondary education. Housing tenure (owner occupier vs. renting) and car ownership (no car, one car and two or more cars) were the two indicators used to measure wealth. Employment status in two categories was included in the model to examine the impact of paid employment at older ages. Family characteristics were measured by household size categorized into three groups: single person household, two person household, and three or more person household.

### Analytical strategy

The analytic strategy included three separate steps. In the first step, the descriptive analysis provided information on distributional characteristics of all variables including the mean time allocated to the various activities across all countries. In the second step, we applied binary logistic regressions to examine the association between time use, SEP, family characteristics and self-reported health simultaneously. The analyses were done separately for men and women as well as pooled models. Estimates in the pooled models were derived from hierarchical modeling of self-reported health in which the variables were added sequentially.

The binary logit model estimated the probability of the dependent variable (self-reported health) to be 1 (Y = 1), which is expressed mathematically as follows:

$$pr(Y=1|x)=\frac{\text{exp}(x\beta )}{1+\text{exp}(x\beta )}$$ (1)

In the third step, a decomposition method was applied to identify the relative contribution of the different factors to total gender inequality in health. We used an extension of the Blinder-Oaxaca decomposition method proposed by Yun [42] for non-linear models to examine the contribution of social factors to female excess in the probability of reporting poor health. The decomposition for a non-linear equation such as *pr*(*Y* = 1) = Φ(Xβ) can be expressed as:

$${\bar{Y}}_{m}-{\bar{Y}}_{w}=\sum_{i=1}^{i=K}W_{\Delta X}^{i}\left[\Phi (X_{m}\beta_{m})-\Phi (X_{w}\beta_{m})\right]+\sum_{i=1}^{i=K}W_{\Delta \beta }^{i}\left[\Phi (X_{w}\beta_{m})-\Phi (X_{w}\beta_{w})\right]$$ (2)

*where* Φ is a standard normal cumulative distribution function, *Y* = health status; β = regression coefficient; X = covariates; *m* = men; *w* = women; *W* = weight assigned to each covariate that is equal to its proportional contribution to the total endowment or coefficient effect.

This decomposition method allows partitioning the health differences between men and women into two components, with men as the reference group [43]. The first component is the *“the endowment effect”* which represents the part of the gender gap in health that is due to differences in group characteristics. The second component is the *“coefficient effect*” which represents the part due to differences in the group processes. In line with Williams [44], we focused on the part of the gap that is due to differences in group characteristics (such as education and time use), with decomposition estimates showing how characteristics contribute individually to the health gap. The contributions of the included factors to the health gap can be positive or negative [45]. Using a counterfactual decomposition framework [42], a positive number indicates a reduction in female excess that would have occurred if women had men’s characteristics. Negative estimates indicate that the variable in question contributes to the gap in the direction that runs counter to the overall health gap [44]. All statistical analyses were performed in STATA version 14 [46].

## Results

### Descriptive statistics

The descriptive statistics for men and women for each country are shown in Tables 1 and 2. Gender differences were found in age, education, wealth, employment status and household size, but there was marked cross-national variation. No gender difference was found in self-reported health in the US and the UK, but in the other countries. Women were slightly older than men. They also had lower educational attainment as compared to men. The largest percentage of elderly men and women who reported having a tertiary education was found in the US (46.9% for men and 40.3% for women), Germany (47.5% for men and 17.5% for women), UK (18.3% for men and 10.0% for women). A larger proportion of women than men reported living in a one-person household.

**Table 1:** General description of the study sample (in percentages, means and SD), men.

	Germany		Italy		Spain		UK		USA	
	(*n* = 1478)		(*n* = 3770)		(*n* = 4234)		(*n* = 1315)		(*n* = 2426)	
	Mean / %	SD	Mean/ %	SD	Mean/ %	SD	Mean/ %	SD	Mean/ %	SD
***Self-reported health***										
Good	39.2%		26.6%		41.5%		52.9%		67.4%	
Poor	60.8%		73.4%		48.5%		47.1%		32.6%	
***Time use Activity***										
Paid work hours/day	0.39	1.66	0.38	1.68	0.22	1.22	0.24	1.30	0.83	2.43
Less than 1	92.8%		94.4%		96.0%		95.5%		87.4%	
1or more	7.2%		5.6%		4.0%		4.5%		12.7%	
House work hours/day	3.62	2.40	2.69	2.48	2.44	2.51	3.79	2.40	2.99	2.77
Less than 4	57.3%		72.3%		74.4%		55.1%		69.9%	
4 to 6	25.8%		17.2%		15.6%		26.1%		14.5%	
>6	16.9%		10.5%		9.9%		18.9%		15.6%	
Active leisure hours/day	4.55	2.68	4.26	2.69	4.17	2.72	3.93	2.90	3.62	3.22
Less than 2	16.3%		20.6%		22.8%		27.5%		37.5%	
2 to 4	31.1%		29.6%		27.2%		31.6%		24.6%	
>4	52.6%		49.9%		50.0%		40.8%		37.9%	
Passive leisure hours/day	3.66	2.03	4.33	2.17	4.51	2.25	4.57	2.50	5.81	3.77
Less than 3	37.9%		26.9%		25.1%		26.9%		23.9%	
3 to 5	40.1%		39.7%		38.8%		35.4%		25.6%	
>5	22.0%		33.5%		36.2%		37.6%		50.6%	
Personal activity hours/day	11.93	1.95	12.71	2.14	12.97	2.35	11.17	1.90	10.89	2.52
Less than 10	12.3%		7.1%		5.4%		23.7%		34.4%	
10 to 12	44.9%		34.0%		30.9%		49.1%		39.9%	
>12	42.8%		59.0%		63.7%		27.2%		25.7%	
***Age***	71.21	4.81	72.50	5.01	72.59	5.09	72.40	5.10	72.89	5.26
65–69	44.1%		35.0%		34.7%		35.4%		33.8%	
70–74	29.6%		29.2%		27.8%		28.1%		24.9%	
75–79	17.1%		19.6%		19.6%		21.3%		20.7%	
80+	9.3%		16.1%		18.0%		15.2%		20.7%	
***Education***										
Incomplete Sec. or less	10.7%		67.5%		68.3%		63.3%		21.5%	
Secondary completed	41.8%		27.7%		23.2%		18.5%		31.7%	
Tertiary Completed or above	47.5%		4.8%		8.5%		18.3%		46.9%	
***Wealth***										
Land tenure										
Renting	41.1%		16.9%		9.4%		27.5%		15.8%	
Owner occupier	58.9%		83.1%		90.7%		72.6%		84.3%	
Car Ownership										
No car	11.8%		16.0%		40.9%		27.9%		-	-
1 Car	72.3%		48.4%		42.0%		57.2%		-	-
2+ Car	16.0%		35.6%		17.1%		14.9%		-	-
***Employment Status***										
Not working for pay	83.7%		92.4%		96.3%		91.8%		77.2%	
Currently in paid employment	16.3%		7.6%		3.7%		8.2%		22.8%	
***Household size***	2.10	0.81	2.36	1.02	2.55	1.20	1.91	0.70	1.77	0.93
1 Member	14.0%		12.9%		10.5%		21.9%		40.6%	
2 Members	70.1%		75.6%		52.3%		69.1%		49.9%	
3+ Members	15.9%		11.5%		37.2%		9.1%		9.6%	

**Table 2:** General description of the study sample (in percentages, means and SD), women.

	Germany		Italy		Spain		UK		USA	
	(*n* = 1848)		(*n* = 4939)		(*n* = 5659)		(*n* = 1694)		(*n* = 4052)	
	Mean / %	SD	Mean / %	SD	Mean / %	SD	Mean / %	SD	Mean / %	SD
***Self-reported health***										
Good	46.8%		16.9%		32.6%		52.9%		68.2%	
Poor	53.2%		83.1%		67.4%		47.1%		31.8%	
***Time use Activity***										
Paid work hours/day	0.09	0.60	0.07	0.71	0.07	0.67	0.09	0.78	0.46	1.80
Less than 1	97.7%		98.9%		98.6%		98.2%		92.6%	
1or more	2.3%		1.2%		1.4%		1.8%		7.4%	
House work hours/day	4.64	2.34	5.14	2.74	4.77	2.71	4.47	2.29	3.79	2.88
Less than 4	38.7%		31.7%		35.8%		40.9%		58.4%	
4 to 6	35.4%		30.3%		32.6%		35.1%		20.4%	
>6	25.8%		38.0%		31.7%		24.1%		21.2%	
Active leisure hours/day	4.15	2.59	2.97	2.21	2.86	2.28	3.63	2.54	3.84	3.09
Less than 2	22.1%		34.3%		37.5%		27.7%		33.1%	
2 to 4	31.6%		38.6%		36.2%		35.8%		25.7%	
>4	46.3%		27.2%		26.3%		36.4%		41.2%	
Passive leisure hours/day	3.39	1.88	3.78	2.10	4.14	2.22	4.22	2.31	4.85	3.35
Less than 3	41.9%		37.5%		31.1%		30.3%		32.9%	
3 to 5	42.4%		38.9%		39.3%		38.9%		26.8%	
>5	15.7%		23.6%		29.6%		30.8%		40.3%	
Personal activity hours/day	11.97	2.03	12.47	2.19	12.57	2.32	11.17	1.96	11.18	2.57
Less than 10	10.2%		8.0%		7.0%		23.0%		29.9%	
10 to 12	47.1%		39.1%		39.6%		48.9%		39.8%	
>12	42.7%		53.0%		53.4%		28.0%		30.3%	
***Age***	71.74	5.13	73.3	5.21	73.24	5.18	73.1	5.00	73.89	5.34
65–69	41.7%		29.5%		30.4%		30.6%		28.0%	
70–74	25.6%		26.9%		27.1%		27.7%		22.5%	
75–79	18.8%		20.7%		19.8%		23.0%		20.8%	
80+	13.9%		22.9%		22.6%		18.7%		28.7%	
***Education***										
Incomplete Sec. or less	28.9%		80.1%		77.7%		76.5%		21.3%	
Secondary completed	53.6%		17.9%		18.5%		13.5%		38.4%	
Tertiary Completed or above	17.5%		2.1%		3.9%		10.0%		40.3%	
***Wealth***										
Land tenure										
Renting	51.7%		23.3%		12.0%		30.8%		21.0%	
Owner occupier	48.3%		76.7%		88.0%		69.2%		79.0%	
Car ownership										
No car	32.4%		38.3%		55.4%		48.6%		-	-
1 Car	59.2%		34.1%		32.1%		43.5%		-	-
2+ Car	8.4%		27.6%		12.5%		7.9%		-	-
***Employment Status***										
Not working for pay	92.4%		98.4%		98.4%		95.7%		86.4%	
Currently in paid employment	7.6%		1.6%		1.6%		4.3%		13.6%	
***Household size***	1.78	1.00	2.00	1.10	2.31	1.30	1.62	0.72	1.52	0.87
1 Member	45.0%		36.6%		26.0%		47.3%		62.3%	
2 Members	42.2%		42.2%		43.2%		46.8%		30.2%	
3+ Members	12.8%		21.3%		30.8%		5.9%		7.5%	

Time use varied considerably between men and women and across countries. Overall, women allocated more time to housework activities compared to men. On the other hand, elderly men tended to devote more time to active leisure, passive leisure and paid work. The cross-country comparison revealed that women in Italy spent on average more time on housework activities (5.1 hours per day). US women spent remarkably fewer hours on housework activities (3.8 hours per day). Time devoted to active leisure did not vary much across countries among men but women. The most time devoted to active leisure was found in Germany (4.1 hours per day), the least active leisure time in Italy (3.0 hours per day) and Spain (2.9 hours per day). Allocation of time for paid work was highest in the US for men and women. Regarding the time allocated to passive leisure, men in the US devoted most hours to these activities (5.8 hours per day), while the lowest value was observed in Germany (3.7 hours per day). Women in all countries spent less time on passive leisure. Finally, the analysis of personal activities showed that men and women in Spain devoted the most time to personal activity (13 and 12.6 hours per day) while the least time spent on these activities was found in the UK and the US (approximately 11.2 hours per day).

### Logistic regression

The results of the multivariate logistic regression models are shown in Tables 3 (pooled model), 4 and 5 (separated by gender and country).

**Table 3:** Multivariate associations between poor self-reported health status, time use, socio-economic position and family characteristics, pooled data of 5 countries. Men and women 65+ years old.

Variables	Model 1^1^	Model 2^2^	Model 3^3^
	aOR (95% CI)	aOR (95% CI)	aOR (95% CI)
***Time use Activity***			
Paid work hours/day			
Less than 1 (ref)			
1or more	0.38 (0.34–0.44)**	1.00 (0.84–1.19)	0.75 (0.63–0.90)**
House work hours/day			
Less than 4 (ref)			
4 to 6	0.93 (0.87–0.99)*	0.86 (0.81–0.92)**	0.76 (0.71–0.81)**
>6	0.92 (0.86–1.00)*	0.83 (0.76–0.90)**	0.65 (0.60–0.71)**
Active leisure hours/day			
Less than 2 (ref)			
2 to 4	0.82 (0.77–0.87)**	0.86 (0.81–0.92)**	0.75 (0.70–0.81)**
>4	0.57 (0.53–0.61)**	0.66 (0.61–0.71)**	0.53 (0.49–0.58)**
Passive leisure hours/day			
Less than 3 (ref)			
3 to 5	1.24 (1.17–1.32)**	1.16 (1.09–1.23)**	1.14 (1.07–1.21)**
>5	1.38 (1.28–1.48)**	1.24 (1.15–1.34)**	1.31 (1.21–1.42)**
Personal activity hours/day			
Less than 10 (ref)			
10 to 12	1.54 (1.43–1.66)**	1.29 (1.19–1.39)**	1.01 (0.94–1.10)
>12	2.84 (2.62–3.07)**	2.05 (1.89–2.23)**	1.43 (1.31–1.56)**
***Sex***			
Men (ref)			
Women		1.20 (1.14–1.27)**	1.32 (1.25–1.40)**
***Age***			
65–69 (ref)			
70–74		1.14 (1.07–1.22)**	1.15 (1.08–1.23)**
75–79		1.35 (1.26–1.45)**	1.41 (1.31–1.52)**
80+		1.32 (1.23–1.42)**	1.44 (1.33–1.55)**
***Education***			
Incomplete Sec. or less (ref)			
Secondary completed		0.47 (0.45–0.50)**	0.58 (0.54–0.61)**
Tertiary completed or above		0.27 (0.25–0.29)**	0.47 (0.43–0.51)**
***Wealth***			
Land tenure			
Renting (ref)			
Owner occupier		0.81 (0.76–0.86)**	0.80 (0.75–0.86)**
***Employment Status***			
Not working for pay (ref)			
Currently in paid employment		0.42 (0.37–0.48)**	0.52 (0.45–0.59)**
***Household size***			
1 member (ref)			
2 members		1.18 (1.11–1.26)**	1.03 (0.97–1.10)
3+ members		1.34 (1.24–1.44)**	1.03 (0.95–1.11)
***Welfare States (countries)***			
Germany (ref)			
Italy			2.85 (2.59–3.14)**
Spain			1.19 (1.09–1.31)**
United Kingdom			0.68 (0.61–0.76)**
United States			0.47 (0.43–0.52)**
Observations	31,425	31,425	31,425
Pseudo R2	0.052	0.107	0.152
Log Likelihood	-20263.22	-19090.029	-18128.073

aOR- adjusted Odd Ratio,

** p<0.01,

* p<0.05. Regression include day-of-week dummies.

^1^ Includes only time use activities

^2^ Includes time use activities, socio-economic position and family characteristics

^3^ Includes time use activities, socio-economic position, family characteristics and countries

**Table 4:** Multivariate associations between poor self-reported health status, time use, socio-economic position and family characteristics. Men, 65+ years old.

Variables	Germany	Italy	Spain	UK	USA
	aOR (95% CI)	aOR (95% CI)	aOR (95% CI)	aOR (95% CI)	aOR (95% CI)
***Time use Activity***					
Paid work hours/day					
Less than 1 (ref)					
1or more	0.77 (0.42–1.41)	0.72 (0.45–1.17)	0.66 (0.42–1.03)*	0.70 (0.33–1.48)	0.63 (0.40–1.00)*
House work hours/day					
Less than 4 (ref)					
4 to 6	0.92 (0.70–1.22)	0.72 (0.58–0.90)**	0.72 (0.60–0.88)**	0.62 (0.46–0.83)**	0.65 (0.49–0.85)**
>6	0.73 (0.50–1.07)	0.98 (0.71–1.34)	0.58 (0.44–0.76)**	0.46 (0.31–0.68)**	0.50 (0.36–0.69)**
Active leisure hours/day					
Less than 2 (ref)					
2 to 4	1.07 (0.76–1.50)	1.07 (0.84–1.36)	0.74 (0.61–0.89)**	0.59 (0.43–0.81)**	0.78 (0.62–0.99)*
>4	0.64 (0.43–0.94)*	0.77 (0.59–1.01)*	0.61(0.50–0.76)**	0.45 (0.31–0.65)**	0.47 (0.36–0.61)**
Passive leisure hours/day					
Less than 3 (ref)					
3 to 5	1.27 (0.98–1.65)	1.12 (0.93–1.35)	1.09 (0.92–1.28)	1.26 (0.93–1.71)	1.13 (0.85–1.50)
>5	1.35 (0.96–1.90)	1.35 (1.07–1.70)*	1.13 (0.93–1.37)	1.16 (0.82–1.64)	1.24 (0.92–1.68)
Personal activity hours/day					
Less than 10 (ref)					
10 to 12	1.22 (0.84–1.78)	0.94 (0.70–1.27)	1.23 (0.91–1.65)	0.80 (0.60–1.09)	1.04 (0.83–1.31)
>12	1.51 (1.01–2.25)**	1.19 (0.87–1.62)	1.91 (1.42–2.58)**	0.99 (0.69–1.41)	1.68 (1.30–2.16)**
***Age***					
65–69 (ref)					
70–74	1.09 (0.84–1.42)	1.77 (1.47–2.13)**	1.14 (0.97–1.34)	0.89 (0.66–1.20)	1.00 (0.78–1.28)
75–79	1.43 (1.04–1.96)*	1.63 (1.32–2.02)**	1.33 (1.11–1.61)**	1.59 (1.15–2.21)**	1.09 (0.84–1.42)
80+	1.23 (0.82–1.85)	2.52 (1.92–3.31)**	1.39 (1.14–1.70)**	1.10 (0.76–1.60)	1.03 (0.79–1.33)
***Education***					
Incomplete Sec. or less (ref)					
Secondary completed	0.78 (0.54–1.14)	0.64 (0.54–0.75)**	0.65 (0.55–0.75)**	0.96 (0.70–1.30)	0.54 (0.43–0.69)**
*Tertiary completed or above*	0.90 (0.62–1.30)	0.60 (0.43–0.84)**	0.48 (0.38–0.61)**	0.72 (0.51–1.01)*	0.40 (0.32–0.51)**
***Wealth***					
Land tenure					
Renting (ref)					
Owner occupier	0.91 (0.72–1.15)	0.92 (0.75–1.14)	0.94 (0.75–1.18)	0.69 (0.52–0.92)*	0.85 (0.66–1.10)
Car ownership					
No car (ref)					
1 car	0.75 (0.53–1.07)	0.67 (0.51–0.88)**	0.72 (0.62–0.83)**	0.71 (0.53–0.95)**	-
2+ cars	1.10 (0.68–1.77)	0.54 (0.40–0.71)**	0.68 (0.55–0.84)**	0.74 (0.47–1.16)	-
***Employment Status***					
Not working for pay (ref)					
Currently in paid employment	0.50 (0.34–0.74)**	0.70 (0.48–1.04)	0.70 (0.44–1.11)	0.38 (0.21–0.68)**	0.54 (0.39–0.75)**
***Household size***					
1 member (ref)					
2 members	1.09 (0.78–1.51)	0.88 (0.68–1.13)	1.00 (0.80–1.25)	0.94 (0.70–1.27)	0.73 (0.60–0.89)**
3 members	1.49 (0.96–2.30)*	0.94 (0.71–1.24)	1.07 (0.84–1.36)	1.95 (1.18–3.24)**	1.02 (0.74–1.41)
Observations	1,478	3,770	4,234	1,315	2,426
Pseudo R2	0.050	0.071	0.063	0.087	0.109
Log Likelihood	-940.8543	-2026.5036	-2693.9923	-830.0513	-1364.4081

aOR- adjusted Odd Ratio; Regression include day-of-week dummies

** p<0.01,

* p<0.05.

**Table 5:** Multivariate associations between poor self-reported health status, socio-economic position, family characteristics and time use. Women, 65+ years old.

Variables	Germany	Italy	Spain	UK	USA
	aOR (95% CI)	aOR (95% CI)	aOR (95% CI)	aOR (95% CI)	aOR (95% CI)
***Time use Activity***					
Paid work hours/day					
Less than 1 (ref)					
1or more	0.49 (0.22–1.05)*	1.48 (0.63–3.48)	0.95(0.48–1.87)	0.86 (0.33–2.20)	1.20 (0.76–1.89)
House work hours/day					
Less than 4 (ref)					
4 to 6	0.85 (0.66–1.08)	1.02 (0.81–1.28)	0.69(0.59–0.81)**	0.85 (0.67–1.10)	0.69 (0.57–0.84)**
>6	0.93 (0.67–1.28)	0.75 (0.57–0.98)*	0.57(0.46–0.69)**	0.70 (0.51–0.96)*	0.62 (0.49–0.79)**
Active leisure hours/day					
Less than 2 (ref)					
2 to 4	0.83 (0.62–1.11)	0.95 (0.78–1.16)	0.63(0.54–0.73)**	0.97 (0.74–1.26)	0.64 (0.53–0.77)**
>4	0.42 (0.30–0.60)**	0.74 (0.57–0.96)*	0.40(0.33–0.49)**	0.86 (0.62–1.19)	0.59 (0.48–0.73)**
Passive leisure hours/day					
Less than 3 (ref)					
3 to 5	1.13 (0.90–1.42)	1.22 (1.02–1.46)*	0.99(0.86–1.14)	1.31 (1.02–1.70)*	1.22 (1.00–1.49)*
>5	1.07 (0.76–1.52)	1.39 (1.06–1.82)*	1.06(0.87–1.28)	2.26 (1.65–3.08)**	1.45 (1.17–1.80)**
Personal activity hours/day					
Less than 10 (ref)					
10 to 12	0.64 (0.46–0.90)*	1.00 (0.76–1.32)	1.07(0.85–1.35)	0.91 (0.69–1.19)	1.17 (0.97–1.41)
>12	0.85 (0.58–1.24)	1.33 (0.98–1.81)*	1.37(1.06–1.76)*	1.44 (1.04–1.99)*	2.05 (1.66–2.53)**
***Age***					
65–69 (ref)					
70–74	0.82 (0.64–1.05)	1.34 (1.11–1.62)**	1.03(0.89–1.20)	1.21 (0.92–1.59)	1.06 (0.85–1.31)
75–79	1.23 (0.93–1.62)	2.33 (1.84–2.96)**	1.23(1.04–1.47)*	1.34 (1.00–1.79)*	1.16 (0.93–1.44)
80+	2.04 (1.46–2.84)**	2.64 (2.02–3.46)**	1.14(0.95–1.37)	1.36 (0.99–1.88)*	1.03 (0.84–1.27)
***Education***					
Incomplete Sec. or less (ref)					
Secondary completed	0.89 (0.70–1.11)	0.66 (0.55–0.79)**	0.47(0.41–0.54)**	1.00 (0.73–1.37)	0.39 (0.32–0.47)**
Tertiary completed or above	0.86 (0.63–1.17)	0.44 (0.28–0.69)**	0.41(0.31–0.55)**	1.35 (0.93–1.95)	0.27 (0.22–0.32)**
***Wealth***					
Land tenure					
Renting (ref)					
Owner occupier	0.79 (0.63–0.97)*	0.98 (0.80–1.18)	1.06(0.88–1.27)	0.68 (0.53–0.87)**	0.62 (0.52–0.74)**
Car ownership					
No car (ref)					
1 car	0.68 (0.53–0.87)**	0.65 (0.52–0.81)**	0.76(0.66–0.88)**	0.53 (0.41–0.69)**	-
2+ cars	0.69 (0.42–1.13)	0.61 (0.49–0.78)**	0.68(0.55–0.85)**	0.23 (0.14–0.39)**	-
***Employment Status***					
Not working for pay (ref)					
Currently in paid employment	0.75 (0.50–1.13)	0.34 (0.17–0.67)**	0.43(0.23–0.81)**	0.64 (0.35–1.18)	0.32 (0.23–0.46)**
***Household size***					
1 member (ref)					
2 members	2.34 (1.83–3.00)**	1.13 (0.92–1.40)	1.05(0.89–1.23)	1.63 (1.27–2.10)**	1.27 (1.07–1.50)**
3 members	1.97 (1.31–2.96)**	1.25 (0.97–1.61)*	1.15(0.94–1.39)	3.83 (2.20–6.69)**	1.04 (0.78–1.39)
Observations	1,848	4,939	5,659	1,694	4,052
Pseudo R2	0.079	0.075	0.071	0.089	0.131
Log Likelihood	-1176.0733	-2077.1254	-3318.7431	-1067.6131	-2201.9978

aOR- adjusted Odd Ratio; Regression include day-of-week dummies

** p<0.01,

* p<0.05.

The pooled model shows that all time use activities were related to health in the crude and the fully adjusted model (Table 3). In the full model, we observed that elderly people who spent more than 1 hour on paid work activities had lower odds of reporting poor health (OR = 0.75; 95% CI = 0.63–0.90) as compared to those who spent less than 1 hour to these activities. Individuals who spent more than 6 hours per day on housework activities had lower odds (OR = 0.65; 95% CI = 0.60–0.71) of reporting poor health compared to those who spent less than 4 hours to these activities. We also observed a strong association between poor health and time devoted to active leisure activities. Individuals who devoted more than 4 hours per day to active leisure activities were less likely to report poor health (OR = 0.53; 95% CI = 0.49–0.58) as compared to those who devoted less than 2 hours per day to these activities. Passive leisure and personal activity (including sleep hours) were associated with higher odds for poor health. Individuals who spent more than 5 hours on passive leisure activities were more likely to report poor health (OR = 1.31; 95% CI = 1.21–1.42) compared to those who devoted less than 3 hours to these activities. The odds of reporting poor health was significantly higher (OR = 1.43; 95% CI = 1.31–1.56) for individuals who spent more than 12 hours per day on personal activities compared to those who spent less than 10 hours.

Regarding the other factors, many patterns were similar to results from other reports. Women were more likely to report poor health than men (OR = 1.32; 95% CI = 1.25–1.40). Educational attainment was significantly associated with health status. We found a negative gradient with the prevalence of poor health increasing with decreasing educational level. Odds of reporting poor health increased with age. Furthermore, the odds of reporting poor health status was lower among homeowners than renters (OR = 0.80; 95% CI = 0.75–0.86). Respondents who were currently in paid employment were less likely to report poor health as compared to those not working for pay (OR = 0.52; 95% CI = 0.45–0.59). Surprisingly, larger household size was positively associated with poor health status in model 2, but this association disappeared in model 3. Compared to Germany, elderly people in Italy and Spain had higher odds of reporting poor health (OR = 2.85; 95% CI = 2.59–3.14 and OR = 1.19; 95% CI = 1.09–1.31), and elderly people in the UK and the US had lower odds of reporting poor health (OR = 0.68; 95% CI = 0.61–0.76 and OR = 0.47; 95% CI = 0.43–0.52).

Tables 4 and 5 show the results of multivariate logistic regression models separately per gender and country.

Among men, time devoted to paid work activities was significantly associated with health in Spain and the US, but not in Germany, Italy and the UK (Table 4). Housework activities and active leisure activities were positively associated with health across the countries, with some inconsistencies in Germany and Italy. With regards to passive leisure and personal activities, we found a negative association with health in some, but not all countries. Here, more time devoted to these activities was associated with poor health.

Among women, only in Germany, paid work was positively associated with health. In contrast, in all countries but Germany, time spent on housework activities was positively associated with health. Time allocated to active leisure was positively associated with health in all countries except the UK. More time spent on passive leisure activities increases the likelihood of reporting poor health among women in Italy, UK and the US, but the effects were not statistically significant in Germany and Spain.

### Non-linear decomposition

Table 6 gives the results of the country specific non-linear decomposition of female excess in the probability of reporting poor health. The female excess in the probability of reporting poor health was statistically decomposed into two parts, namely the part of inequality due to differences in group characteristics (endowment effect), and the part of inequality due to differences in group processes (coefficient effects). As discussed in the method section, we focused on the part of inequality due to differences in group characteristics (by variables) and the overall inequality due to differences in group processes.

**Table 6:** Non-linear decomposition of female excess in the probability of reporting poor health, by country.

Inequality contributions in terms of differences in group characteristics (by variables) & group processes	Germany		Italy		Spain		UK*	USA*
	Absolute (95% CI)	Percent	Absolute (95% CI)	Percent	Absolute (95% CI)	Percent	Absolute (95% CI)	Absolute (95% CI)
Predicted mean in women	0.532 (0.510–0.555)		0.831 (0.820–0.841)		0.674 (0.662–0.686)		0.470 (0.447–0.494)	0.318 (0.304–0.332)
Predicted mean in men	0.392 (0.368–0.417)		0.734 (0.720–0.749)		0.585 (0.570–0.599)		0.471 (0.444–0.498)	0.326 (0.307–0.344)
**Female excess**	**0.140 (0.106–0.174)**		**0.096 (0.079–0.114)**		**0.089 (0.070–0.108)**		**-0.000 (-0.036–0.036)**	**-0.008 (-0.031–0.016)**
Age	0.003 (0.001–0.006)	2.30%	0.011 (0.008–0.014)	11.10%	0.003 (0.001–0.004)	2.80%	0.003 (-0.001–0.007)	0.000 (-0.001–0.001)
Education	0.016 (0.005–0.028)	11.50%	0.010 (0.007–0.013)	10.10%	0.016 (0.012–0.019)	17.50%	0.002 (-0.004–0.009)	0.003 (-0.004–0.010)
Land tenure	0.010 (0.000–0.021)	7.50%	0.013 (0.009–0.017)	13.40%	0.009 (0.006–0.013)	10.50%	0.026 (0.003–0.049)	0.002 (-0.002–0.005)
Car	0.005 (0.001–0.009)	3.40%	0.001 (-0.001–0.002)	0.80%	0.000 (-0.001–0.001)	0.20%	0.003 (-0.001–0.008)	-
Employment status	0.010 (0.004–0.016)	7.20%	0.006 (0.002–0.009)	5.80%	0.002 (-0.000–0.004)	2.10%	0.006 (-0.002–0.013)	0.007 (-0.006–0.020)
Household Size	-0.021 (-0.031–-0.010)	-14.70%	0.001 (-0.004–0.005)	0.80%	-0.001 (-0.005–0.003)	-1.00%	-0.024 (-0.042–-0.007)	-0.001 (-0.003–0.001)
Paidwork	0.009 (0.001–0.017)	6.70%	0.001 (-0.006–0.007)	0.60%	0.001 (-0.003–0.004)	0.80%	0.003 (-0.002–0.008)	0.001 (-0.002–0.004)
Housework	-0.009 (-0.026–0.009)	-6.10%	-0.000 (-0.045–0.044)	-0.50%	0.002 (-0.040–0.044)	2.20%	-0.022 (-0.038–-0.006)	-0.007 (-0.020–0.005)
Active leisure	0.013 (0.004–0.022)	9.10%	0.013 (-0.011–0.037)	13.40%	0.017 (-0.007–0.041)	18.80%	0.007 (-0.001–0.015)	-0.002 (-0.005–0.001)
Passive leisure	-0.000 (-0.005–0.004)	-0.20%	-0.006 (-0.016–0.005)	-5.80%	-0.007 (-0.014–0.001)	-7.30%	-0.007 (-0.013–-0.001)	-0.002 (-0.007–0.003)
Personal activity	0.000 (-0.001–0.002)	0.30%	-0.003 (-0.007–0.002)	-2.60%	-0.015 (-0.023–-0.007)	-17.00%	0.000 (-0.001–0.001)	0.002 (-0.002–0.006)
Contribution to that part of inequality due differences in group characteristics **(Endowment effects)**	0.038 (0.020–0.056)	**27.00%**	0.045 (0.035–0.056)	**47.20%**	0.026 (0.016–0.037)	**29.70%**	-0.003 (-0.019–0.013)	0.003 (-0.007–0.014)
Contribution to that part of inequality due differences in group processes **(Coefficient effects)**	0.102 (0.073–0.131)	73.00%	0.051 (0.037–0.065)	52.80%	0.063 (0.047–0.079)	70.30%	0.003 (-0.029–0.035)	-0.011 (-0.032–0.010)

* No female excess in the probability of reporting poor health

CI: 95 percent confidence interval.

In absolute terms, Germany reported the lowest and Italy the highest predicted probability in poor health. In contrast, Germany reported the highest female excess (0.140; 95% CI = 0.106–0.174) in the probability of reporting poor health followed by Italy (0.096; 95% CI = 0.079–0.114) and Spain (0.089; 95% CI = 0.070–0.108) while no female excess was found in the UK and the US.

Italy reported the highest total gender gap (approximately 47%) attributed to differences in group characteristics, followed by Spain (approximately 30%) and Germany (approximately 27%). The two largest contributing factors to this component of gender inequality in health among elderly people across all countries are education and active leisure. If women were to allocate the same time to active leisure activities as men, the female excess in the probability of reporting poor health would be reduced by approximately 18% in Spain and approximately 13% in Italy. In Germany, education is the largest contributor to the part of the inequality deriving from differences in group characteristics. The gender gap would be reduced by approximately 12% if women had the same educational attainment as men. Passive leisure contributed negatively to this part of inequality in all countries, and personal activities showed mixed contributions in different countries.

## Discussion

As far as we know, this is the first study to analyze simultaneously the relationship between time use activities, SEP, household characteristics and health among elderly men and women in four European countries and the US. Our study also examined gender and cross-country differences in patterns of time use among the elderly. On the descriptive level, our study showed that elderly women allocate more time to housework activities as compared to men. Elderly men tended to devote more time to active leisure, passive leisure and paid work with some cross-country variations. All time use activities were related to health with paid work, housework and active leisure activities positively and passive leisure and personal activities negatively associated with health. However, the magnitude of the association varied by gender and across countries. We found gender differences in health, but these differences vary visibly across countries with no gender gap in health observable in the UK and the US. Decomposing the gap in health, the study showed that differences in the time allocated to active leisure and level of educational attainment accounted for the largest share of the health gap.

### Gender differences in health status

Our findings could only partially confirm the conventional view on the health paradox in contemporary welfare countries [47], that women live longer yet they report poorer health [48,49]. The results indicated that gender differences in health among the elderly do not exist in the UK and the US. Other studies have also found no gender differences in self-reported health and a number of health conditions in countries such as Finland [50], the UK and the US. In the UK, Macintyre et al. [47] revealed that female excess varied according to health conditions and phase of the life cycle. They concluded that female excess in reporting poor health was consistently found across the life span for psychological distress conditions, but was far less apparent or reversed for a number of physical symptoms and conditions. Likewise, a longitudinal study in the US among men and women in their late adult life found that women had better self-reported health than men, but they report higher rates of symptoms related to discomfort and functional impairment than men [51].

Explanations for the existence or the non-existence of the gender gap in health refer to differences in labor force participation, education, recreational activities, and domestic activities among men and women [47]. The results of this present study are consistent with these explanations and confirm that differences in the levels of educational attainment as well as active leisure activities largely explain the gender differences in health among elderly. In the UK and the US, we found relatively little differences in the highest educational level among men and women, as shown Tables 1 and 2.

### Gender differences in socio-demographic, economic position and family characteristics related to health status

Prior evidence demonstrated that health inequality based on social class exists among the elderly [52,53]. Gender disparities in socio-demographic and economic positions have been suggested as a possible explanation for these observed differences in health [17–19], but there may be variations in gender differences in vulnerability to socio-economic status indicators on health conditions across countries [54,55]. All the three indicators of SEP including education, wealth and employment status showed consistent patterns of health disparities between groups of high and low SEP in our study. However, our results showed also significant gender and cross-country differences in the magnitude of the associations, similar to those reported in previous studies among the elderly [54,55]. Age was significantly associated with poor health among men and women in all countries but not in the US. Nonetheless, previous research also showed a strong negative association between age and health among the elderly [56]. In line with previous studies [19,21], educational attainment was significantly associated with health status in both elderly men and women in all countries; the prevalence of poor health increased with decreasing educational level.

In all the countries, the labour force participation among the elderly was lower for women than for men (Tables 1 and 2). The US recorded the highest proportion (approximately 23% for men and approximately 14% for women) of labour force participation compared to the European countries in our study. Evidence from the US suggests that changes in pension systems and a high cost of health care in recent years are potential explanations for the high employment rate among the elderly [57], whereas most EU countries have universal health care systems [40].

Housing tenure and car ownership were used as proxy indicators for measuring wealth. These two indicators were significantly associated with health among men and women, but not in all countries observed. For example, we found a strong positive association with health among male owner occupiers in the UK, which could not be observed in the other countries. Meanwhile, among women, this strong association persisted not only in the UK but also Germany and the US. This results concurred with the findings of Dalstra et al. [21], who found that health differences for housing tenure were generally smaller in all countries observed except the UK and the Netherlands. Our findings suggest that the relationship between housing tenure and health among the elderly largely depends on the country and the findings may be due to the differences in the national housing policies and local housing market. We further showed a strong positive association of car ownership with health for both elderly men and women. This result is in line with a study conducted by Arber and Ginn [58], which have shown that elderly people who do not own a car have higher odds of reporting poor health. However, we found a weaker association for men in Germany, but not elsewhere, while the association was stronger for women in Germany and the other countries. These findings suggest that wealth plays a critical role in the health of elderly men and women and could be linked to the fact that material resources such as car ownership enable the elderly to carry out daily activities such as shopping with ease. Also, material resources enable older adults to participate in leisure and social activities [58], as these activities were found in this study and previous studies [59,60] to be associated with good health among elderly men and women.

Surprisingly, our findings regarding household size suggest that living in a larger household is associated with poor health among elderly men and men. However, the association was stronger among women than men, except in Italy. Among men, the association was not significant in the southern European countries. A common explanation for this phenomenon is that people living in larger households, especially women, suffer more stress [61].

### Gender differences in time use activities related to health status

Regarding time spent on housework activities, the results showed that there were gender and cross-country differences. Women spent more time than men in housework activities, consistent with prior evidence [62,63]. Nonetheless, time allocated to housework activities among men has increased over the years [62], and has become more equal with women over time [63]. Despite the apparent gender inequality in time allocation to housework activities, we found that time devoted to housework activities was positively associated with health among both women and men across all countries. Among the working population, unequal division of household labor has been linked with adverse health outcomes among women [24,64]. This could to some extent be explained by the “double burden” of work hypothesis, where the combination of paid market work and domestic work has been proposed be more stressful for women than men [65]. However, given a changed time availability after retirement, this hypothesis might lose its premise in explaining the effect of housework activities on health among elderly men and women because the elderly may not have the same time constraints of combining both paid work and household activities like the working-age adults.

The few studies that examined the effects of housework on the health among elderly men have given inconsistent results [66–68]. The study by Lawlor et al. [67] examined the impact of domestic activities on health among British women aged 60 to 79 years. Heavy housework activities were not associated with reduced likelihoods of being overweight. Although Wen et al. [68] found the association of housework and health of older adults to be not significant overall, washing clothes and house cleaning were negatively associated with health among women. They speculated that these activities involve almost no interpersonal communication and less extensive physical exertion, hence the negative association with health. On the other hand, housework that involves physical activity may be beneficial to health among older adults [68]. Our measures of housework includes, beside washing clothes and cleaning house, activities that require some form of physical exertion such as gardening, grocery shopping, odd jobs and domestic travels (S1 Table). Accordingly, our finding of the effects of housework on health among elderly men and women may be influenced by the extent of physical exertion of these activities.

Paid work at older ages was associated with good health among men, as found in previous studies [69–71], but the patterns were not consistent among women. While we found a negative gradient for paid work and poor health, a longitudinal evidence by Luoh and Herzog [71] suggest that a moderate amount of time devoted to paid work activities is sufficient for a good health and additional time spent on paid work activities will not necessarily increase the health benefit among the elderly.

Active leisure was positively associated with health status for both elderly men and women in all countries, consistent with prior evidence [59,60,72]. Nonetheless, allocation of time to active leisure activities differed between men and women and across countries. As shown in other studies, men devoted more time to active leisure than women [73–75]. Again, consistent with previous studies [36,76] we found that women in Italy and Spain devoted less time to active leisure activities as compared to their female counterparts in other countries. Burda et al. [77] suggested that cross-country differences in social norms with regards to total work distribution and time use patterns are possible explanations for the patterns of gender inequality observed in the Southern European countries. In these countries, gender roles often are shaped in a traditional way where housework activities are traditionally reserved for women [27,78]. Thus, it is evident from our results that part of the time that would be devoted to active leisure was reallocated to housework activities. As a consequence, women in these countries spent a relatively shorter amount of time on active leisure activities while the amount of time devoted to housework activities increases.

Nevertheless, it seems that regardless of cultural or social norms time devoted to active leisure activities is positively related to health of the elderly. Previous studies have also stressed the importance of older adult’s participation in active leisure activities for social, psychological and physical health benefit. For example, a study by Kim et al. [60] found that active leisure activities increase the psychological feelings and physical functioning among the elderly. Also, more time spent on fitness activities has been found to be associated with lower risk of mortality among the elderly [79].

In contrast to active leisure activities, passive leisure activities such as listening to radio and tapes, watching television and relaxing were negatively associated with health among both men and women. Similar to our study, television viewing has been linked to poor health, cognitive decline, depressive symptoms and anxiety in older men and women [80,81]. Furthermore, an increase in time spent on television has also be found to be associated with a varied series of health outcomes among older men and women including obesity [82] and cardiovascular disease [83]. However, Nguyen et al. [84] found that television viewing among older adults was a strategy to cope with depressive symptoms. Likewise, Potts and Sanchez [85] found that television viewing is a way of escaping from depression. The negative association of passive leisure activities on health in most previous studies and this study can be explained by the sedentary nature of these activities as they require little or no physical or mental energy.

Similar to passive leisure activities, more time devoted to personal activities such as sleep, meals and personal services was negatively associated with health among elderly in all countries. No difference in time devoted to personal activities was found between men and women within-countries, but there were cross-national variations. Our results also showed that elderly men and women spent more time on personal activities than those observed for the other activities but more of this time was devoted to sleep. This is not surprising because the increasing incidence of health conditions at older age restrict daily activities among the elderly [1]. As a result, time devoted to personal activities, especially sleep increases among older men and women. Sleep duration has been found to be associated with adverse health outcomes among the elderly including obesity and sarcopenia [86], and cardiovascular disease [87]. Ikehara et al. [87] showed a U-shaped association between sleep duration and mortality from cardiovascular diseases and non-cardiovascular diseases among older adults. In our study, we are unable to separate out the effect of only sleep duration on health. However, our measure of personal activities which includes not only time devoted to sleep, but also time spent on meals and personal services such as bathing concurred with these findings, suggesting that longer durations of these activities are associated with poor health among elderly men and women.

### Policy and research implications

Our findings provide evidence of the relationship between social roles (time allocated to role-related activities) and health among the elderly in a gender-specific and country-comparative context. We compared data from Germany, Spain, Italy, UK and the US. These countries represent different institutional settings and differ on national policies and social norms [27]. National policies, in particular welfare provisions, have been shown to have a significant effect on health [88]. Therefore, gender and cross-national variations in health may be explained to some extent by national context and social norms. This complex relationship between the state and family may increase or decrease cost and time in terms of social support, provision of care services and leisure opportunities. As a consequence, men and women may display different time allocation patterns in response to the existing welfare system. In the specific case of our comparative analysis, we found that cross-national variations in welfare provisions of care such as service and cash benefit for the elderly [89] may explain to some extent the differences in the patterns of time use. Furthermore, cultural norms may also shape the relationship between time use allocation decision and health as found in the southern European countries [27], where gender roles are still shaped in a more traditional way. As a result, women devote more time to housework. This time inflexible routine housework activities limit opportunities for engaging in other social and leisure activities for women [28], which affects their health negatively. In our decomposition analysis, we found that the share of health inequality that is explained by active leisure is more than the share due to SEP in Italy and Spain. Therefore, the results of this study demonstrate the importance of taking into account time spent in social roles in the analysis of gender differences in health among the elderly.

## Limitations

The cross-sectional design of this study prevents us to conclude any causal association. Because the association between time use activities and health may be reciprocal, conclusions such as “older people allocate less time to certain time use activities due to poor health” (and vice versa) cannot be drawn. Again, this study relied on self-reports of time use activities and general health status. Nonetheless estimates from time use surveys have been found to be more accurate and reliable than survey estimates [90,91]. Although self-reported health has been shown to be an inclusive and accurate measure of health status [92], and a good predictor of mortality among the elderly [93], it should also be acknowledged that there may be gender and cross-country differences in reporting [94,95].

Another possible limitation is that only primary activities were considered in the analysis due to data limitations, although it has been shown that performing secondary activities like care activities and watching television simultaneously with primary activities may provide some detailed information about time use. Thus, eliminating parallel activities may distort the picture of the time devoted to the various task of life. However, in practice, secondary activities are usually ignored in time-use analysis [96]. Due to data constraints, this study could not include income which is a traditional measure of SEP. Albeit, in research among the elderly, wealth has been suggested as a better measure of economic well-being as compared to income [97], as it better reflects the permanent economic position [98]. Therefore, the present study utilizes car ownership and land tenure as proxy indicators for measuring wealth [21,58]. Although we have controlled for a variety of confounding factors, biological traits and behavioral risk factors which may play a role in the explanation of gender inequalities in health were not included in the present findings.

Despite these limitations, this study provides the first overview of time use activities and their relationship with health using a large-scale and comparative set of time use data across Europe and the US of the elderly population.

## Conclusions

The overall goal of this study was to explain the gender differences in health among the elderly by taking time use activities and other social factors into account. We conclude that education and time spent on active leisure are the largest contributors to gender differences in health among the elderly. The evidence provided in this study demonstrates the need for and usefulness of an integrated framework of social factors in analyzing and explaining the gender and cross-national differences in health among the elderly.

## Supporting information

S1 Table Typology of activities. (DOCX)
