# Supplementary material for: Gender inequality in self-reported health among the elderly in contemporary welfare countries: A cross-country analysis of time use activities, socioeconomic positions and family characteristics
Source: PLoS One. 2017 Sep 26;12(9):e0184676. doi: 10.1371/journal.pone.0184676 (PMC5614435; doi:10.1371/journal.pone.0184676)
Supplement: S1 Table — (DOCX) [file pone.0184676.s001.docx]

|  | | |  |  |  |  |  |
| --- | --- | --- | --- | --- | --- | --- | --- |
| Broad categories of activity | | |  | **Name of variable** **(harmonised)** | | **Description** | |
| 1. Paid work | | |  | AV01 | | Paid work | |
|  | | |  | Av02 | | Paid work at home | |
|  |  |  |  | AV03 | | Second job | |
|  |  |  |  | AV05 | | Travel to/ from work | |
|  |  |  |  |  |  |  |  |
| 2.Housework | | |  | AV06 | | Cooking/Washing up | |
|  |  |  |  | AV07 | | Housework | |
|  |  |  |  | AV08 | | Odd jobs | |
|  |  |  |  | AV09 | | Gardening, pets | |
|  |  |  |  | AV10 | | Shopping | |
|  |  |  |  | AV12 | | Domestic travel | |
|  |  |  |  |  |  |  |  |
| 3.Active leisure |  |  |  | AV11 | | Child care | |
|  |  |  |  | AV23 | | Civic duties | |
|  |  |  |  | AV19 | | Active sport | |
|  |  |  |  | AV21 | | Walks | |
|  |  |  |  | AV17 | | Leisure travel | |
|  |  |  |  | AV18 | | Excursions | |
|  |  |  |  | AV22 | | Religious activities | |
|  |  |  |  | AV24 | | Cinema, theatre | |
|  |  |  |  | AV26 | | Social club | |
|  |  |  |  | AV27 | | Pub | |
|  |  |  |  | AV28 | | Restaurant | |
|  |  |  |  | AV29 | | Visiting friends | |
|  |  |  |  | AV04 | | School/classes | |
|  |  |  |  | AV20 | | Passive/observer sports | |
|  |  |  |  | AV33 | | Study | |
|  |  |  |  | AV34 | | Reading books | |
|  |  |  |  | AV35 | | Reading papers and magazines | |
|  |  |  |  | AV37 | | Conversation | |
|  |  |  |  | AV38 | | Entertaining friends | |
|  |  |  |  | AV39 | | Knitting sewing etc. | |
|  |  |  |  | AV40 | | Other hobbies | |
|  |  |  |  |  |  |  |  |
| 4.Passive leisure |  |  |  | AV30 | | Listening to radio | |
|  |  |  |  | AV31 | | Television, video | |
|  |  |  |  | AV32 | | Listening to tapes etc. | |
|  |  |  |  | AV36 | | Relaxing | |
|  |  |  |  |  |  |  |  |
| 5.Personal activity |  |  |  | AV13 | | Dressing/toilet | |
|  |  |  |  | AV14 | | Personal Services | |
|  |  |  |  | AV15 | | Meals, snacks | |
|  |  |  |  | AV16 | | Sleep | |

**S1 Table. Typology of activities**
